# Supplementary material for: Anatomy of leaf apical hydathodes in four monocotyledon plants of economic and academic relevance
Source: PLoS One. 2020 Sep 17;15(9):e0232566. doi: 10.1371/journal.pone.0232566 (PMC7498026; doi:10.1371/journal.pone.0232566)
Supplement: S1 Fig — Observation of pit membranes integrity in hydathodes of maize (A-B), rice (C-D), Brachypodium (E-F) and sugarcane (G) by transmission electron microscopy. (PDF) [file pone.0232566.s001.pdf]

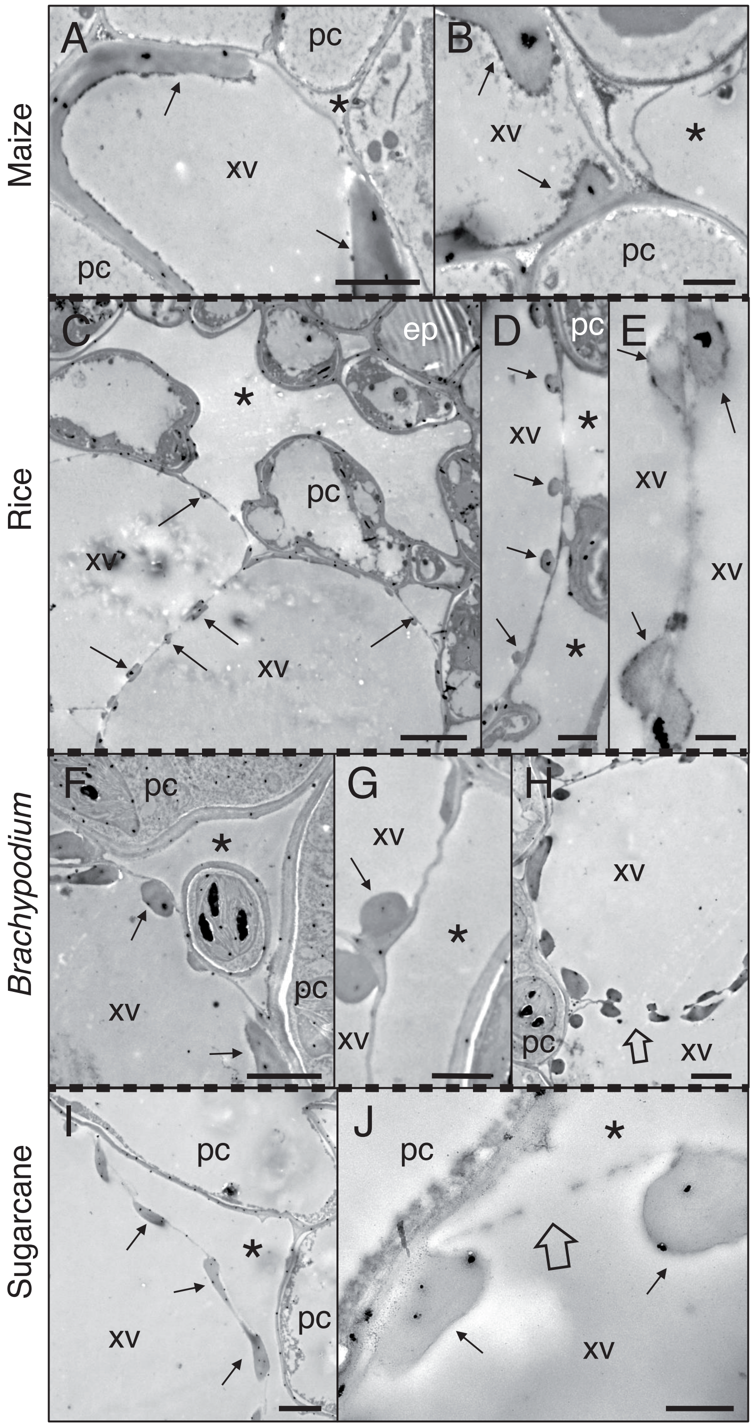

S1 Fig. Observation of pit membranes integrity in hydathodes of maize (A-B), rice (C-E), *Brachypodium* (F-H) and sugarcane (I-J) by transmission electron microscopy. Ultra-thin sections were treated with PATAg to stain for polysaccharides and enhance contrast. Lignified ornamentations of xylem vessels (xv) are shown by arrows. Pit membranes corresponds to thin cell walls bridging xylem vessel ornamentations. Note the absence of PATAg-reactive material between ornamentations from vessel to vessel in panels H and J (open arrows). Legends: epithem cell (ep); parenchyma cell (pc); vascular element (xv); inter-cellular space and meatuses (asterisk).Scale bars: F = 5 µm; A, C, E, H = 2 µm; B, D, G and I = 1 µm; J= 0.5 µm.

S1 Figure

Jauneau et al.
